# Molecular and functional evaluation of a novel HIF inhibitor, benzopyranyl 1,2,3-triazole compound

**DOI:** 10.18632/oncotarget.13955

**Published:** 2016-12-15

**Authors:** Kyunghye Park, Hye Eun Lee, Sun Hee Lee, Doohyun Lee, Taeho Lee, You Mie Lee

**Affiliations:** ^1^ BK21 Plus KNU Multi-Omics based Creative Drug Research Team, National Basic Research Laboratory of Vascular Homeostasis Regulation, Kyungpook National University, Buk-gu, 702-701, Daegu, Republic of Korea; ^2^ College of Pharmacy, Research Institute of Pharmaceutical Sciences, Kyungpook National University, Buk-gu, 702-701, Daegu, Republic of Korea

**Keywords:** HIF-1α inhibitor, chemical library, benzopyranyl 1,2,3-triazole

## Abstract

Hypoxia occurs in a variety of pathological events, including the formation of solid tumors. Hypoxia-inducible factor (HIF)-1α is stabilized under hypoxic conditions and is a key molecule in tumor growth and angiogenesis. Seeking to develop novel cancer therapeutics, we investigated small molecules from our in-house chemical libraries to target HIF-1α. We employed a dual-luciferase assay that uses a luciferase (Luc) reporter vector harboring five copies of hypoxia-responsive element (HRE) in the promoter. Under hypoxic conditions that increased Luc reporter activity by four-fold, we screened 144 different compounds, nine of which showed 30–50% inhibition of hypoxia-induced Luc reporter activity. Among these, “Compound 12, a benzopyranyl 1,2,3-triazole” was the most efficient at inhibiting the expression of HIF-1α under hypoxic conditions, reducing its expression by 80%. Under hypoxic conditions, the half maximal IC_50_ of the compound was 24 nM in HEK-293 human embryonic kidney cells, and 2 nM in A549 human lung carcinoma cells. Under hypoxic conditions, Compound 12 increased hydroxylated HIF-1α levels and HIF-1α ubiquitination, and also dose-dependently decreased HIF-1α target gene expression as well as vascular endothelial growth factor (VEGF) secretion. Furthermore, this compound inhibited VEGF-induced *in vitro* angiogenesis in human umbilical vein endothelial cells (HUVECs), and *in vivo*, it inhibited chick chorioallantoic membrane angiogenesis. In allogaft assays, cotreatment with Compound 12 and gefitinib significantly inhibited tumor growth and angiogenesis. Compound 12 can be a novel inhibitor of HIF-1α by accelerating its degradation, and shows much potential as an anti-cancer agent through its ability to suppress tumor growth and angiogenesis.

## INTRODUCTION

Hypoxia is defined as a condition of lower O_2_, usually ≤ 2% O_2_, while anoxia (severe hypoxia) is defined as a condition of ≤ 0.02% O_2_. Most mammalian tissues experience 2–9% O_2_ (on average, a partial pressure of 40 mm Hg), and ambient air is 21% O_2_ (a partial pressure of 150 mm Hg). Hypoxia occurs in a variety of pathological events such as in arthritic joints, stroke, inflammation, tissue ischemia, and the growth of solid tumors. Under these circumstances, hypoxic cells generate energy through glycolysis to minimize oxygen consumption, and they activate signaling pathways that mediate cell growth, angiogenesis, and anti-apoptosis to improve cell survival [1–3]. Solid tumors contain hypoxic regions due to rapid cell proliferation, and hypoxia is a strong stimulator of tumor angiogenesis [4]. Hypoxia research has provided considerable information on the molecular mechanisms by which O_2_ levels influence the properties and behaviors of tumors, including altered cellular metabolism, proliferation, and angiogenesis, as well as increased resistance to chemotherapy and radiation [1, 5].

Hypoxia-inducible factors (HIFs) are well known as master regulators of O_2_ homeostasis, and they mediate many transcriptional changes in response to low O_2_ tension [6]. HIF-1 is expressed by all extant metazoan organisms. HIF-1 consists of an O_2_-sensitive α subunit and a constitutively expressed β subunit [7]. HIF-1α is constantly synthesized, but under normoxic conditions, it is rapidly degraded. Prolyl hydroxylase domain enzymes (PHDs) hydroxylate two proline residues (402 and/or 564) within the O_2_-dependent degradation (ODD) domain of HIF-1α. Subsequently, HIF-1α binds to the von Hippel-Lindau tumor suppressor protein (pVHL) and recruits an E3 ubiquitin ligase, resulting in HIF-1α ubiquitination and proteasomal degradation [7, 8]. Under hypoxic conditions, however, PHD activity is attenuated, leading to HIF-1α protein stabilization. Stabilized HIF-1α dimerizes with HIF-1β and translocate into the nucleus. The HIF-1α/β heterodimer binds to hypoxia response elements (HREs) of target gene promoters, and consequently activates the transcription of downstream genes involved in angiogenesis, metastasis, apoptosis, and glycolysis [9–11].

Increased HIF-1 activity could result in increased cell survival or angiogenesis during hypoxia. However, decreased HIF-1 activity could inhibit cell survival or reduce the angiogenic activity of hypoxic regions such as those found in solid tumors. For these reasons, HIF-1 could be an important drug target for several diseases such as cancer, stroke, and heart disease, in which hypoxia is a central aspect [12]. In the process of adapting to a hypoxic environment, tumor cells promote the transcription of genes associated with angiogenesis, metabolism, cell proliferation, survival, pH regulation, and cell migration. Many of these genes are regulated by HIF-1α; therefore, HIF-1α has emerged as an attractive target for the development of novel cancer therapeutics [13, 14]. For this reason, we screened small molecules to find potent HIF-1α inhibitors, using reporter gene assays. We found that the candidate with the strongest HIF-1α-inhibiting activity was a benzopyranyl 1,2,3-triazole compound, so we examined its biological activities. We determined that its inhibitory effect is achieved via increasing HIF-1α degradation by ubiquitination. We then confirmed this benzopyranyl 1,2,3-triazole compound's effects on angiogenesis and tumor progression *in vivo*, at the molecular as well as the histological level.

## RESULTS

### Selection of candidate HIF-1α inhibitors from in-house chemical library via HRE reporter assay

Because HIF-1α is an attractive target for the development of cancer therapeutics, we screened small molecules to find HIF-1α inhibitors. To test cellular toxicity, we performed MTT assays at various concentrations (0.5–5 μM) of the 144 compounds (3–146). Compound 22 was too toxic at every dose, so we excluded Compound 22 from further study. Some Compounds such as 51, 53, and 58 showed cell toxicity at the highest dose (5 μM) (Supplementary Figure S1), so we omitted this concentration from further investigation.

**Figure 1:**
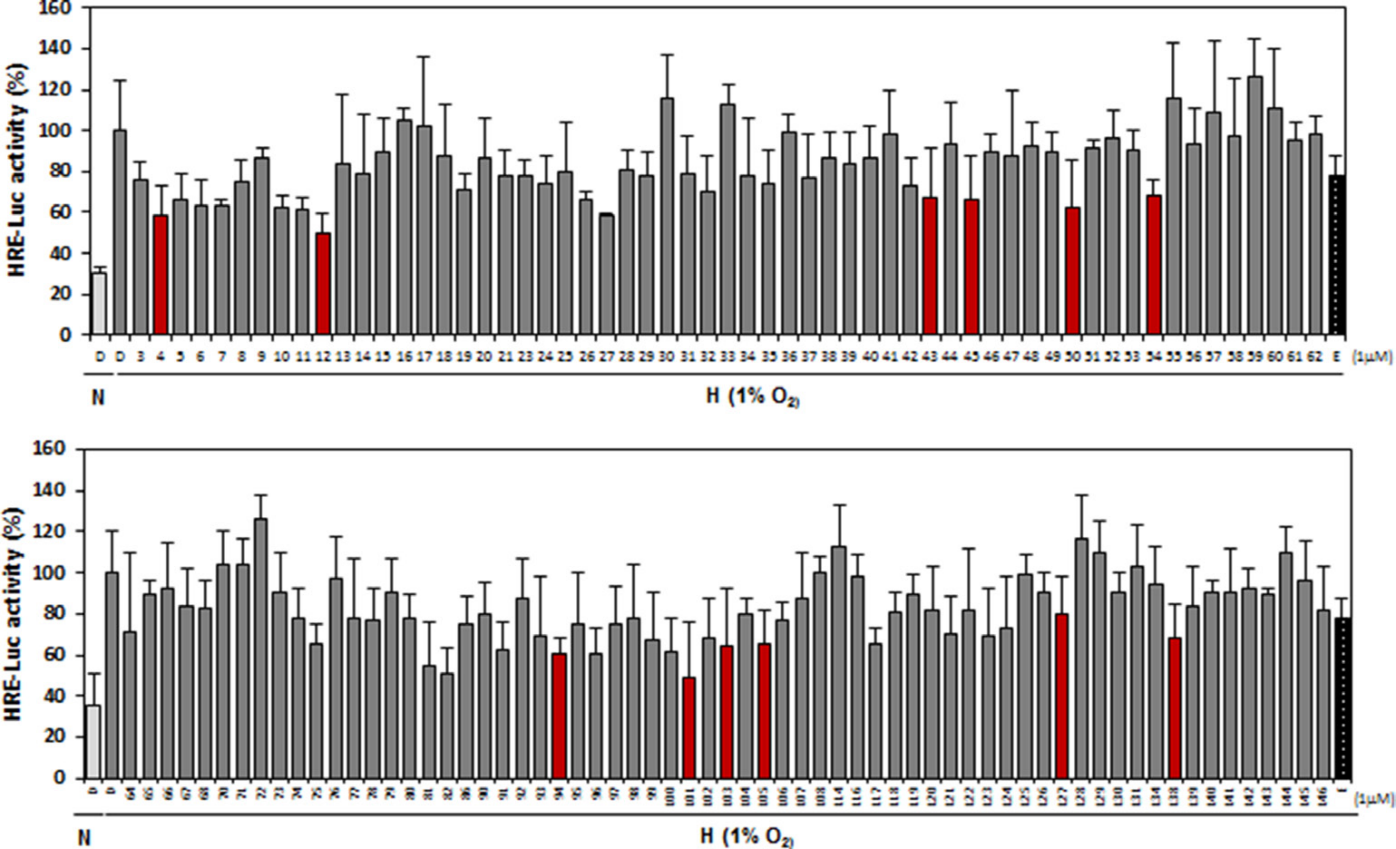
Screening of 144 compounds from an in-house chemical library for inhibition of HIF-1 activity HEK-293 cells were co-transfected with luciferase reporter plasmids (pGL3-HRE-luciferase) and Renilla vector (pRL-SV40). Cells were exposed to hypoxic conditions in the presence or absence of chemicals for 24 h, and were then lysed for assays of luciferase activity. Echinomycin (E), a known HIF-1α inhibitor, was used as a positive control. N, normoxia, H, hypoxia. Three independent experiments were performed in triplicate.

To identify active inhibitors of HIF-1α, a cell-based reporter assay was performed. HEK-293 cells were co-transfected with pGL3-5xHRE-Luc plasmid harboring five copies of hypoxia responsive element (HRE) from the VEGF gene promoter, and with pRL-SV40 encoding Renilla genes. The HRE-containing promoter of the HIF-1 target genes is regulated by HIF-1 activation. Transfected cells were treated with chemical compounds at 1 μM and were exposed to hypoxic conditions (1% O_2_) for 24 h, and then were assayed. Echinomycin (E), a well-known HIF-1α inhibitor, was used as a positive control. Compared to normoxic control conditions, hypoxia increased this luciferase reporter's activity by approximately three-fold. Among the Compounds tested, nine (12, 45, 54, 94, 101, 103, 105, 127 and 138) showed effects of significantly decreasing reporter activity (30–50% inhibition compared to hypoxic control) (Figure 1) and were selected for further investigation.

### Compound 12 is selected as the most potent inhibitor of HIF-1α protein expression in the screened chemical library

**Figure 2:**
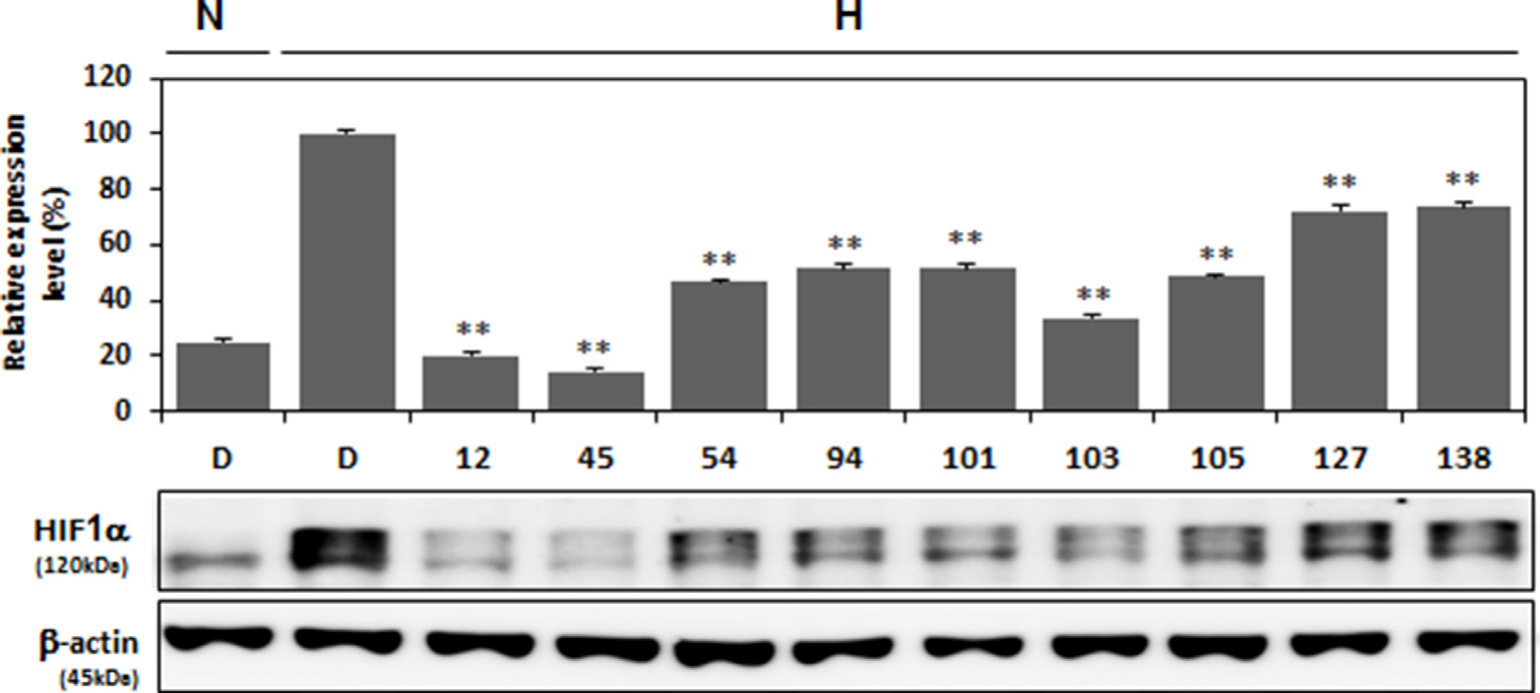
Decreased expression of HIF-1α protein under hypoxic conditions by nine selected compounds A549 cells were treated with nine compounds (each at 1 μM concentration) that had been effective in inhibiting HRE luciferase reporter activity (results shown in Figure 1) (red bar). Treatment was under hypoxic conditions for 16 h. Cells were then lysed for western blot analysis, which was conduced with antibodies against HIF-1α and β-actin. Expression levels were quantified by ImageJ (NIH, Bethesda, MO, USA) and graphed. **P < 0.01 versus 1% O_2_ DMSO (D)-treated control group.

Next, we evaluated the inhibitory effects of the nine aforementioned test compounds on HIF-1α protein accumulation by western blot analyses in A549 cells experiencing hypoxic conditions. A549 cells were treated with 1 μM of each compound for 16 h at hypoxic conditions, and all nine compounds were found to significantly downregulate HIF-1α expression. In particular, Compounds 12 and 45 showed the most potent inhibitory effect on HIF-1α accumulation (Figure 2); however, because the inhibitory effects of Compound 45 in the reporter assay were less than those of Compound 12 (Figure 1), we chose Compound 12 for further study.

**Figure 3:**
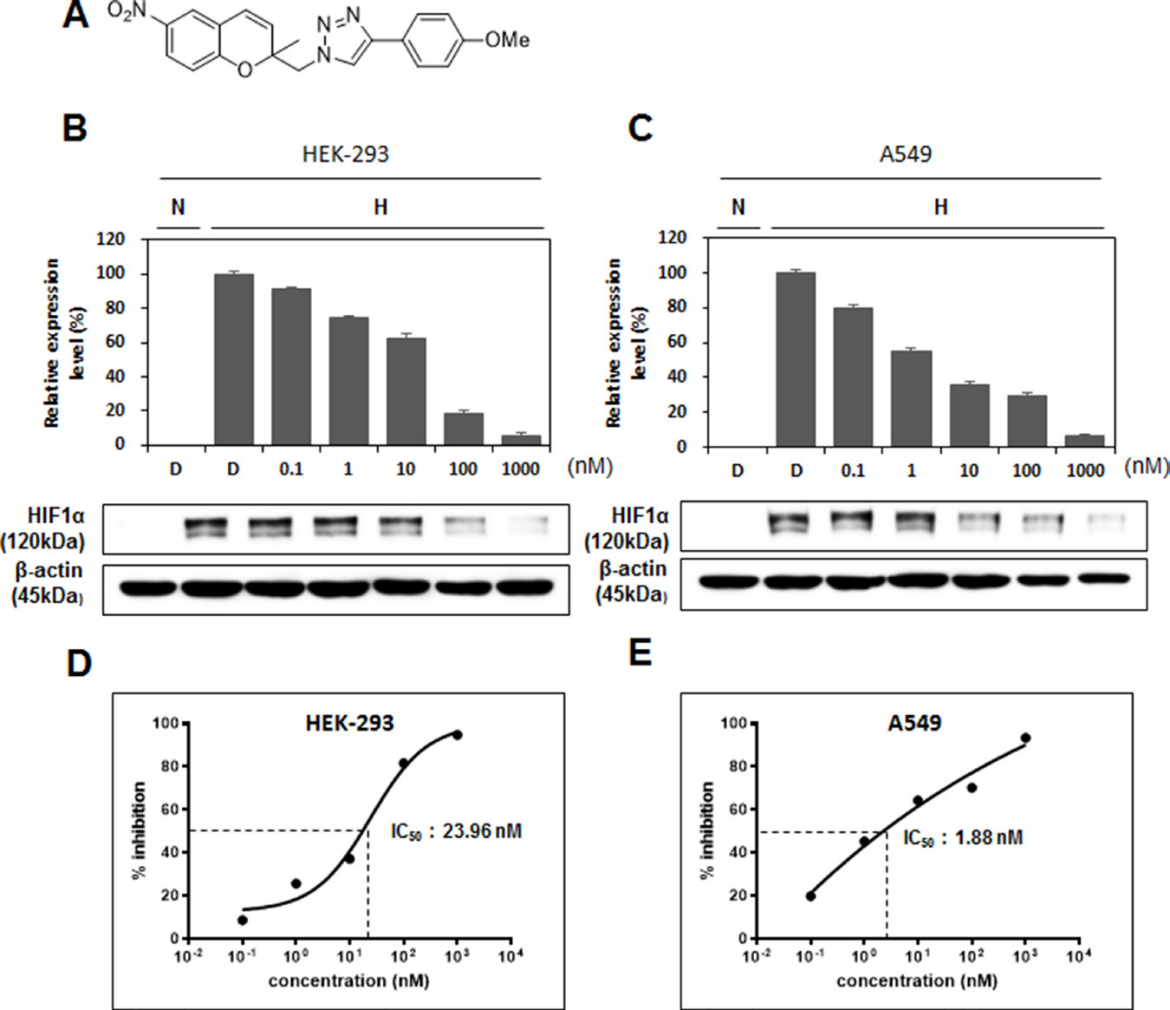
Chemical structure of Compound 12 and its dose-dependent inhibitory effects on HIF-1α expression (**A**) Chemical structure of Compound 12: 4-(4-methoxyphenyl)-1-((2-methyl-6-nitro-2*H*-chromen-2-yl)methyl)-1*H*-1,2,3-triazole. (**B**, **C**) After HEK-293 cells (B) and A549 cells (C) were treated with Compound 12 (at concentrations of 0 nM, 0.1 nM, 1 nM, 10 nM, 100 nM, and 1000 nM) under hypoxic conditions for 16 h, expression of HIF-1α was determined by western blot analysis and then graphed. (**D**, **E**) Half maximal inhibitory concentration (IC_50_) values were calculated using GraphPad Prism software, using the quantified HIF-1α levels determined in B and C.

As shown in Figure 3A, the chemical structure of Compound 12 shows that it is a benzopyranyl 1,2,3-triazole. This compound was synthesized by Cu(I)-catalyzed [3 + 2] cycloaddition of 2-(azidomethyl)-2-methyl-6-nitro-2*H*-chromene and 1-ethynyl-4-methoxybenzene [15, 16]. Compound 12 has a benzopyran scaffold structure that is novel when compared to the chemical structures of known HIF-1α inhibitors, such as YC-1 [3-(5′-hydroxymethyl-2′-furyl)-1-benzyl indazole] [17], topotecan (*S*)-10-[(dimethylamino)methyl]-4-ethyl-4,9-dihydroxy-1*H*-pyrano[3′,4′:6,7]indolizino[1,2-*b*]quinoline-3,14(4*H*,12*H*)-dione monohydrochloride [18], echinomycin [19], and manassantin [20]. Based on our results (shown in Figures 1 and 2) and the known chemical structures (Supplementary Figure S2) of the compounds we tested in Figure 2, we can speculate why Compound 12 behaves uniquely among the 145 compounds of our chemical library. We hypothesize that its activity is due to both the 6-nitro-benzopyran and the 1,4-disubstituted 1,2,3-triazole moiety—because, among the compounds shown in Figure 2 as having an inhibitory activity on HIF-1α expression, Compounds 12, 54, and 138 were 6-nitro-benzopyran derivatives, and Compounds 12, 94, 101, 103, and 105 have the 1,4-disubstituted 1,2,3-triazole moiety (Supplementary Figure S2).

At 1 μM concentration, Compound 12 suppressed HIF-1α transactivation activity by 50%, and HIF-1α protein levels by 80%, when compared to the hypoxic control group (Figures 1 and 2). Therefore, we decided to test Compound 12 at concentrations lower than 1 μM, to determine the minimum effective concentration needed to achieve inhibition of HIF-1α. HEK-293 cells and A549 cells were treated with Compound 12, at 0.1–1000 nM concentrations, under hypoxic conditions for 16 h. Surprisingly, even at 1 nM concentration, Compound 12 downregulated HIF-1α protein expression by approx. 30% in HEK-293 cells, and by approx. 50% in A549 cells (Figure 3B and 3C). GraphPad Prism software was used to calculate the values of half maximal effective concentrations (EC_50_); we determined that the half maximal inhibitory concentrations (IC_50_) of Compound 12 necessary for the inhibition of HIF-1α protein levels in HEK-293 cells and A549 cells were 23.96 nM and 1.88 nM, respectively (Figure 3D and 3E). When compared to other HIF-1α inhibitors, such as YC-1 (2–50 μM) [21], LW6 (2.5–3 μM) [22], echinomycin (1.2 nM), bortezomib (0.6–30 nM) [23], Apigenin (10–90 μM) [24], and recently developed SYP-5 (10 μM) [25]. Compound 12 has a significantly potent inhibitory effect on HIF-1α protein levels.

### Compound 12 inhibits HIF-1α protein stability via increased ubiquitination

**Figure 4:**
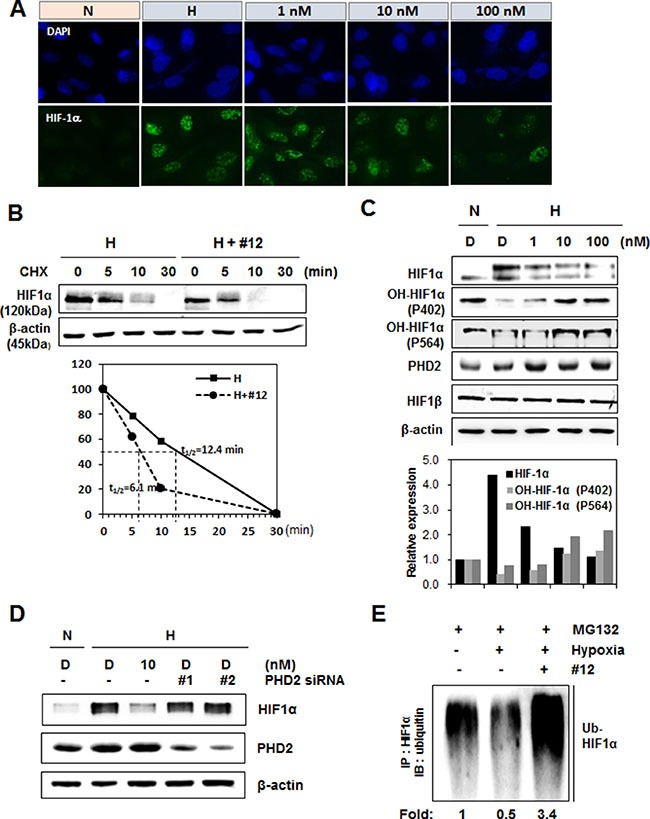
Compound 12 increased hydroxylation and proteasome-mediated degradation of HIF-1α (**A**) A549 cells were treated with Compound 12 (at concentrations of 0 nM, 1 nM, 10 nM, and 100 nM) for 16 h. Cells were fixed with paraformaldehyde; an immunofluorescence assay was then performed using anti-HIF-1α antibodies and Alexa Fluor 488-labeled secondary antibodies (green). Nuclei were stained with DAPI. N, normoxia; H, hypoxia. (**B**) A549 cells were treated with Compound 12 (10 nM concentration) and exposed to hypoxic conditions for 16 h. Cells were immediately treated with cycloheximide (CHX, 100 μg/mL) for the time indicated, and HIF-1α protein levels were determined by western blotting. β-actin was used as an internal control. Half-life (t_1/2_) of HIF-1α protein was determined using relative expression levels of HIF-1α. (**C**) A549 cells were treated with Compound 12 (at concentrations of 0 nM, 1 nM, 10 nM, and 100 nM) for 16 h. The expression levels of HIF-1α, hydroxylated (OH)-HIF-1α at P402 or P564, and PHD2 were determined via western blotting using specific antibodies. N, normoxia; H, hypoxia. (**D**) A549 cells were transfected with two kinds of small interfering RNAs targeting PHD2 and then treated with Compound 12 under hypoxic exposure for 16 h. Cell lysates were immunoblotted with an anti-HIF-1α and PHD2 antibody. (**E**) After A549 cells were treated with Compound 12 for 16 h, cells were treated with MG132 (20 μM) for 4 h. Cell lysates were immunoprecipitated (IP) with anti-HIF-1α antibody, and were then immunoblotted (IB) with anti-ubiquitin antibody. Relative levels of ubiquitinated HIF-1α were quantified and graphed.

To further confirm the effects of Compound 12 on the inhibition of HIF-1α protein expression, we performed an immunofluorescent assay in A549 cells. Cells were treated with Compound 12 (1–100 nM) for 16 h under hypoxic conditions, and were then fixed with paraformaldehyde. Fixed cells were processed to allow immunostaining of HIF-1α by a HIF-1α antibody. Under hypoxic conditions, untreated cells showed HIF-1α accumulated in the nucleus; by contrast, under the same conditions, cells treated with Compound 12 showed that accumulation of nuclear HIF-1α had been inhibited in dose-dependent manner. However, this did not affect the subcellular localization of HIF-1α (Figure 4A). This suggests that Compound 12 does not induce cytosolic translocation of HIF-1α, but rather reduces the protein's stability.

We then determined the half-life of HIF-1α in the presence or absence of Compound 12. A549 cells were treated with Compound 12 at 10 nM, under hypoxic conditions for 16 h, and then were immediately treated with cycloheximide (CHX, an inhibitor of protein synthesis). The half-life (t_1/2_) of HIF-1α protein was 12.4 min in control cells, but in the presence of Compound 12, HIF-1α was degraded two times faster than control cells; i.e., t_1/2_ of HIF-1α was 6.1 min (Figure 4B), indicating that Compound 12 promotes HIF-1α protein degradation.

Because in the O_2_-dependent degradation pathway PHDs hydroxylate the proline residues of HIF-1α in its O_2_-dependent degradation (ODD) domain [26], expression of hydroxylated (OH)-HIF-1α at proline 402 and 564, and PHD2 was investigated. Compound 12 increased hydroxylation of HIF-1α in a dose-dependent manner, and PHD2 levels were increased even at the lowest dose (1 nM) of Compound 12, suggesting that Compound 12 regulates HIF-1α degradation via increased hydroxylation of the HIF-1α ODD domain (Figure 4C). We also determined that Compound 12 did not inhibit HIF-1β expression (Figure 4C). To further confirm the involvement of PHD2 in HIF-1α degradation by Compound 12, two different small interfering (si) RNA of PHD2 were used. Decreased expression of HIF-1α by Compound 12 under hypoxic conditions was recovered by knockdown of PHD2 (Figure 4D). Next, to check whether Compound 12 changes the ubiquitination status of HIF-1α, cells were treated with MG132 (a proteasome inhibitor) while under hypoxic conditions, and in the presence or absence of Compound 12. Cell lysates were immunoprecipitated (IP) with HIF-1α antibody, and then were immunoblotted (IB) with anti-ubiquitin antibody. Compared to normoxic conditions, ubiquitination of HIF-1α under hypoxic conditions (and in the absence of Compound 12) clearly decreased; however, in the presence of Compound 12, ubiquitination of HIF-1α under hypoxic conditions significantly increased, compared to normoxic control cells (Figure 4E). These results suggest that Compound 12 inhibits HIF-1α protein stability by promoting HIF-1α protein degradation via increased proline hydroxylation and subsequent ubiquitination.

### Compound 12 suppresses HIF-1α target gene expression and angiogenesis

**Figure 5:**
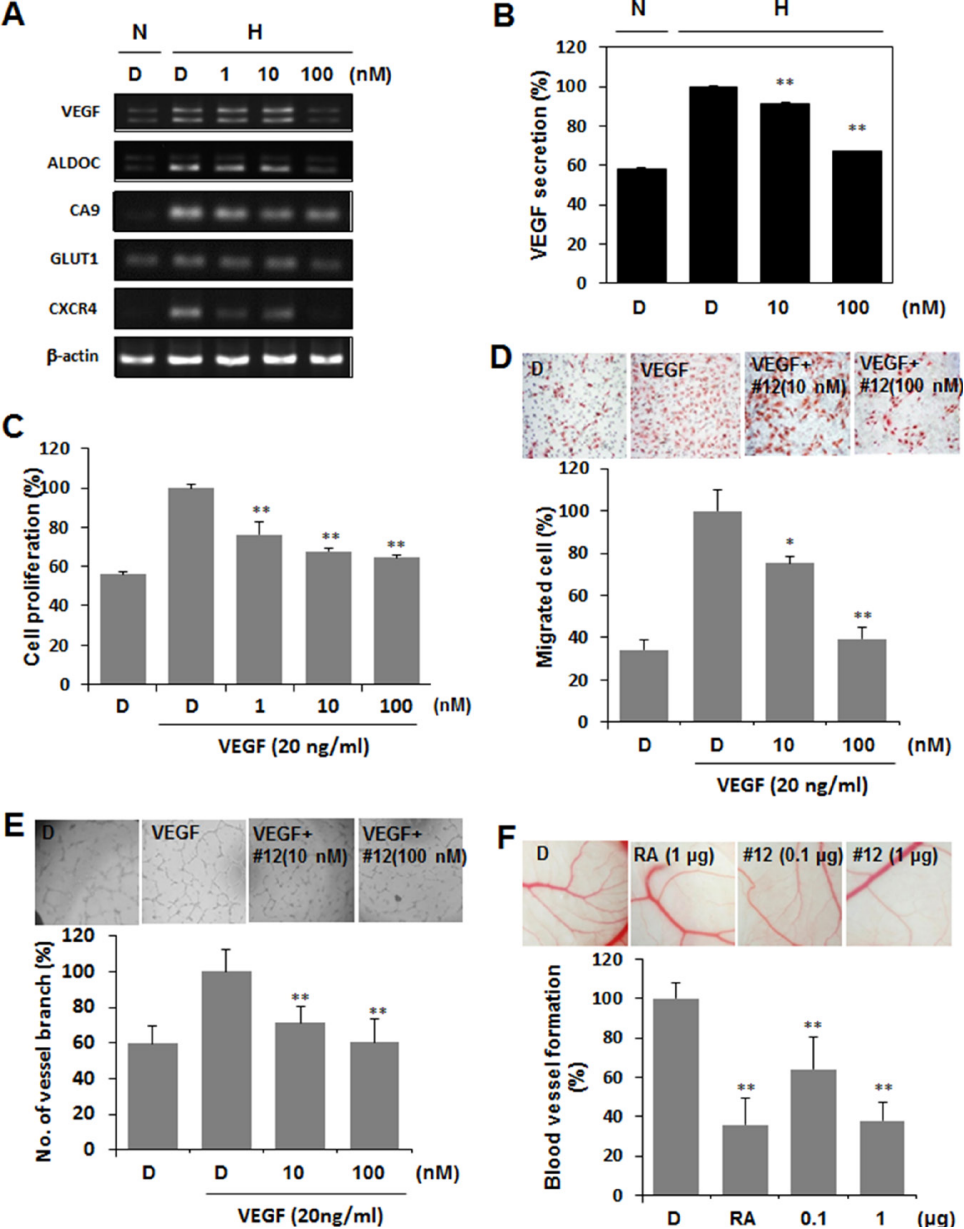
Compound 12 suppressed HIF-1α target gene expression, and confirmation of in vitro results via determination of effects on angiogenesis in vivo (**A**) A549 cells were treated with Compound 12 (at concentrations of 0 nM, 1 nM, 10 nM, and 100 nM) for 16 h. The mRNA levels of HIF-1α target genes were determined by semiquantitative RT-PCR. β-actin was used as an internal control. D. DMSO vehicle control. N, normoxia; H, hypoxia. (**B**) A549 cells were treated with serum-reduced culture media containing Compound 12 (at concentrations of 0 nM, 10 nM, and 100 nM) for 16 h. Media supernatant was collected, and secreted VEGF was determined by ELISA after removal of cellular debris. N, normoxia; H, hypoxia. **P < 0.01 versus hypoxia (H) DMSO (D)-treated control. (**C**) HUVECs were treated with Compound 12, at the indicated concentrations, in the presence of VEGF (20 ng/mL) for 24 h in 1% serum-containing media. BrdU proliferation assays were then performed. **P < 0.01 versus VEGF-treated DMSO (D) group. (**D**). HUVECs were seeded into the upper chamber of the transwell; VEGF (20 ng/mL) and Compound 12, at the indicated concentrations, were then added to the lower chamber, and the transwell was incubated at 37°C for 24 h. Cells that migrated through the membrane (8 μm pore size) were stained with hematoxylin and eosin, pictured as shown in upper panel, and counted (graph). *P < 0.05, **P < 0.01 versus VEGF-treated control. (**E**) HUVECs were seeded on Matrigel-coated 96 well plates and treated with Compound 12, at the indicated concentrations, in the presence of VEGF (20 ng/mL) for 24 h. Changes in cellular morphology were observed under a microscope and photographed at 100× magnification. Statistical significance: *P < 0.05, **P < 0.01 versus VEGF treated control. (**F**) Compound 12 (0.1 μg and 1 μg dosages) and retinoic acid (RA, positive control) were applied to the ED 4.5 CAM for two days, neovessel formation from the large vessels was observed, and percentages of positive angiogenic eggs (from total eggs tested) were calculated.

As HIF-1α regulates transcription of its target genes, which are involved in angiogenesis, glucose metabolism, and metastasis in tumor progression [27, 28], we determined the effects of Compound 12 on HIF-1 target gene expression at the mRNA level by semiquantitative RT-PCR. As expected, Compound 12 suppressed the mRNA levels of VEGF, aldolase C (ALDOC), carbonic anhydrase 9 (CA9), glucose transporter 1 (GLUT1), and chemokine receptor type 4 (CXCR4), in a dose-dependent manner (Figure 5A). Among these, VEGF is a secreted protein and is one of the most potent contributors to tumor angiogenesis and metastasis. Thus, we checked secreted VEGF protein levels in culture media via ELISA. In cells under hypoxic conditions for 16 h (but in the absence of Compound 12), secreted VEGF levels were increased by 1.7-fold when compared to normoxic conditions; however, Compound 12 significantly suppressed hypoxia-induced VEGF secretion, in a dose-dependent manner (Figure 5B).

The inhibitory effects of Compound 12 on VEGF secretion motivated us to investigate whether it also suppressed VEGF-induced angiogenesis. During the process of angiogenesis, vascular endothelial cells proliferate, migrate into avascular regions, and mature into the vessel structure [29]. To check this process, we cultured HUVECs and determined cell proliferation using the BrdU uptake assay. Serum-starved HUVECs were treated with Compound 12 in the presence or absence of 20 ng/mL VEGF for 16 h. VEGF increased endothelial cell proliferation by 1.8-fold, but Compound 12 significantly inhibited VEGF-induced proliferation, in did so in a dose-dependent manner (Figure 5C). Endothelial migration and tube formation abilities induced by VEGF were also significantly inhibited by treatment with Compound 12 (Figure 5D and 5E). To confirm these *in vitro* results *in vivo*, we performed a chick CAM assay. Similar to retinoic acid (RA, the positive control), Compound 12 significantly suppressed CAM angiogenesis, in a dose-dependent manner (Figure 5F).

### Cotreatment of Compound 12 with a chemotherapeutic agent suppresses allogaft tumor growth and angiogenesis

**Figure 6:**
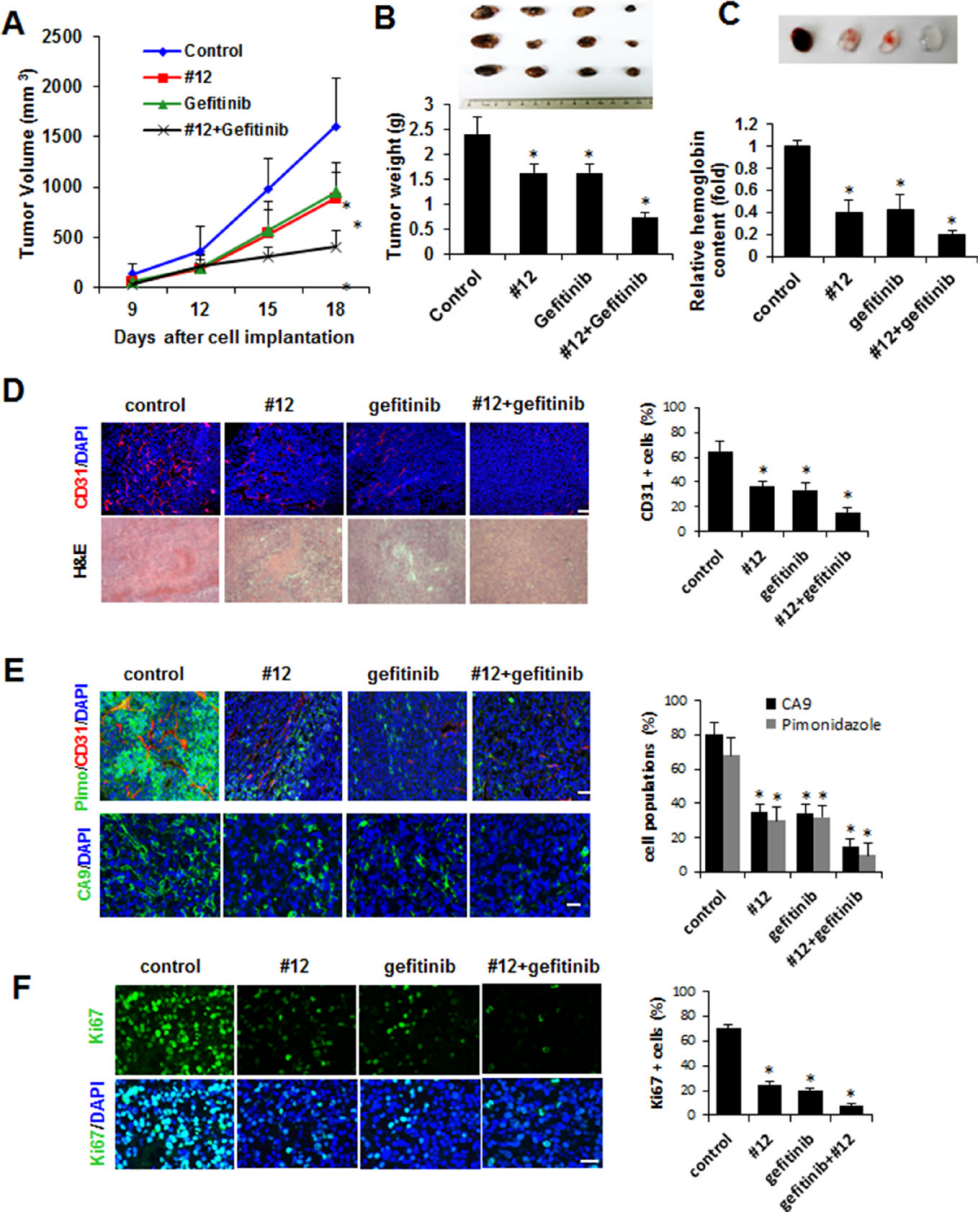
Compound 12 suppressed allogaft tumor growth and enhanced the effects of a chemotherapeutic agent (**A**, **B**) Lewis lung carcinoma cells were inoculated into flanks of C57BL/6J mice (Day 0). From two days after inoculation, mice were treated intraperitoneally with Compound 12 (5 mg/kg body weight) and gefitinib (50 mg/kg body weight), three times a week, for 18 days and then sacrificed. Tumor size (A) and tumor weight (B) were measured three times a week and on the final day, respectively. Tumor masses were photographed and shown in box below the graph. **P < 0.05 versus control group. (**C**) Matrigel was treated with DMSO, Compound 12 (100 nM), and gefitinib (5 μM) and inoculated in the flank of C57BL/6J mice. After 10 days matrigel plug was removed and photographed. The content of hemoglobin was quantified and graphed. (**D**) Tumor tissue sections (10 μm) indicated were stained with H&E and immunostained with anti-CD31 antibody to detect microvessel density; CD31+ cell percentages were then calculated and graphed. Scale bar, 100 μm. **P < 0.05 versus control group. (**E**) Images and quantitative comparisons (graph) for hypoxic regions, immunostained CA9 protein, and microvessels (CD31+ cells) in tumor center regions are shown. Scale bar, 100 μm. **P < 0.05 versus control group. (**F**) Images and quantitative comparisons (graph) for Ki67+ proliferating cells in tumor center regions are shown. Scale bar, 100 μm. **P < 0.05 versus control group.

HIF-1α is synthesized by growth factor signaling pathways, such as those of EGFR [30] [31]. Inhibition of HIF-1α in its pathways of synthesis, as well as in its pathway of degradation, would seem to make for a more efficient anti-cancer/anti-angiogenesis treatment. To confirm this, we performed a tumor allogaft experiment using Lewis lung carcinoma (LLC) cells. 1 × 10^6^ LLC cells were inoculated into the mouse flank subcutaneously (s.c.), and treatment with Compound 12 (5 mg/kg) and/or gefitinib (an EGFR inhibitor, 50 mg/kg), via intraperitoneal (i.p.) administration, was performed every other day for 20 days. At Day 20, mice were sacrificed and tumor tissue was fixed in formaldehyde for 1 h for further histological examination. Individually, gefitinib alone and Compound 12 alone both inhibited tumor growth significantly; administered as a combination, however, they suppressed tumor growth more effectively than did either drug alone (Figure 6A). During the entire treatment period, no noticeable alteration of mice body weight was observed in all groups (Supplementary Figure S3). As shown in Figure 6B, tumor weight was significantly decreased in the Compound 12-treated group, and to an extent, similar or identical to the gefitinib-treated group. To confirm the effect of Compound 12 on angiogenesis, we analyzed angiogenesis using the Matrigel plug assay. As expected, anti-angiogenic activity was increased in tumors treated either with Compound 12 or with gefitinib (Figure 6C). To confirm the effects of Compound 12 on anti-angiogenesis, we sectioned the tumor tissues to determine microvessel density (MSD) via immunohistochemistry (using CD31 antibody). MSD in the vehicle-treated control tumor mass was very high, but treatment with Compound 12 or gefitinib significantly decreased MSD. Cotreatment with Compound 12 and gefitinib synergistically decreased MSD (Figure 6D).

We determined levels of hypoxia in tissues via: 1) antibodies against the hypoxic cell marker pimonidazole (PIMO), and 2) checking expression levels of carbonic anhydrase (CA9), a downstream target of HIF-1. We found that levels of both PIMO and CA9 were significantly diminished in tumors treated with either Compound 12 alone or gefitinib alone, and that PIMO/CA9 levels had decreased synergistically in the group treated with the combination of Compound 12 plus gefitinib (Figure 6E). It is likely that cells expressing the Ki-67 protein (a marker for cellular proliferation) were also significantly decreased in tumors treated either with Compound 12 or with gefitinib (Figure 6F). Taken together, these results suggest that Compound 12, a benzopyranyl 1,2,3-triazole compound, is a novel HIF-1α inhibitor. It seems to have suppressed HIF-1α stability and activity in cancer cells, and to have synergistically inhibited tumor growth when combined with other chemotherapeutic agents. These results suggest that this benzopyranyl 1,2,3-triazole compound can be used in combination therapies, with other targeted chemotherapeutic agents, to inhibit tumor growth and intratumoral angiogenesis.

## DISCUSSION

To improve cancer therapy, manipulation of the tumor microenvironment has been continuously investigated. The responses of tumors to radiation and chemotherapeutic agents depend on factors in the tumor microenvironment, including tumor cell-extracellular matrix interaction [32], tumor-immune cell interaction [33, 34], and tumor oxygenation [34]. In the 1970’s, Folkman proposed the concept of targeting the blood vessels in tumors, to starve the tumor of oxygen and nutrients [35]; however, hypoxia increases HIF-1α and its target, VEGF, resulting in increased blood vessels in the tumor [36]. However, as tumor vasculatures have leaky and chaotic structures, hypoxic regions are generated again as tumors grow [37]. In turn, HIF-1α is overexpressed by recurrent hypoxia, causing increased activity of HIF target genes involved in angiogenesis, metabolism, metastasis, and radioresistance, and chemoresistance [34, 38]. In addition, targeting angiogenesis via treatment with VEGF monoclonal antibodies increases the hypoxic region, again resulting in HIF-1α accumulation and consequential increases in the expression of HIF target genes such as VEGF [39, 40]. Therefore, the collective effects of targeting HIF-1α have more potential for inhibiting tumor growth than does targeting angiogenesis alone. Here we suggest that 5 mg/kg body weight dose of HIF-1α inhibitor, Compound 12 in combination therapy, 1/10 of gefitinib, because of possible toxicity but it was sufficient to be effective (Figure 6). However, owing to the complexity of the HIF-1 transcriptome, inhibition of HIF-1α may also result in unpredictable outcomes in human patients [41]. Among known HIF-1α inhibitors, Campothecin has topoisomerase I inhibitory activity [42] and PX-12 (1-methylpropyl 2-imidazolyl disulfide) inhibits thioredoxin-1 (Trx-1) activity [43], suggesting the probability of Compound 12 having other unspecific effect besides HIF-1α inhibitory activity. Furthermore, the powerful selection occurring in cancer cells exposed to several independent, targeted therapies suggests that administration of multiple agents simultaneously is essential for the successful treatment of human cancer [44–46]. Therefore, the use of HIF-1α inhibitors as anticancer agents must be adopted within the overall strategy of combination therapy [41]. We used gefitinib to inhibit EGFR, and Compound 12 to inhibit HIF-1α. We determined that inhibition of EGFR functions to decrease both HIF-1α synthesis and VEGF expression. Surprisingly, erlotinib—another EGFR inhibitor—decreases hypoxic regions in tumor tissues and induces vascular normalization in tumors, which, in turn, improves the efficacies of chemotherapy and radiotherapy [47]. The results of our allogaft experiments showed that the combination of Compound 12 and gefitinib decreased the size of the hypoxic region and of the tumor itself through increased apoptosis and decreased angiogenesis. From the erlotinib study [47], we can speculate that Compound 12 might enhance the effects of gefitinib not only by inhibiting HIF-1α synthesis, but also by inducing vascular normalization, resulting in improved vascular delivery of gefitinib to the tumor tissues. The effects of Compound 12 on tumor vascular normalization in spontaneous or allogaft tumors and the mechanisms by which these effects are achieved should be further investigated.

The IC_50_ of Compound 12 for the inhibition of HIF-1α protein levels in the hypoxic cells we used was around 2–24 nM. As the IC_50_ values of other HIF-1α inhibitors are 2–50 μM for YC-1 [21], 2.5–3 μM for LW6 [22], 10–90 μM for Apigenin [24], and 10 μM for SYP-5 [25], it is clear that Compound 12 is a far more potent inhibitor of HIF-1α activity. Another HIF-1α inhibitor, Bortezomib inhibits p300-HIF-1α binding at nanomolar concentrations (0.6–30 nM), almost equivalent to Compound 12 to repress HIF-1α protein expression and nuclear accumulation by inhibiting both PI3K/Akt/mTOR and MAPK pathways in prostate cancer cells [23]. Treatments using Compound 12 at nanomolar concentrations could easily be combined with other targeted therapies such as treatment with gefitinib, Herceptin (trastuzumab), and other medicines. In conclusion, Compound 12, a benzopyranyl 1,2,3-triazole, demonstrates strong inhibitory activity against HIF-1α stability and transactivation, thereby showing both anti-angiogenic and anti-tumor activity. Furthermore, the addition of gefitinib enhanced the effects of Compound 12 on anti-tumor activity by decreasing hypoxia-mediated tumor growth. Investigations to confirm the direct target(s) of Compound 12, and to develop simpler and more potent benzopyranyl 1,2,3-triazole analogues, should be undertaken.

## MATERIALS AND METHODS

### Materials

One hundred forty-four small molecules not previously evaluated for their pharmacological effects were obtained from our in-house chemical library [15, 16, 48, 49] (Kyungpook National University, College of Pharmacy, Laboratory of Combinatorial and Medicinal Chemistry) and screened for HIF-1α inhibition. Chemical compounds were dissolved in dimethyl sulfoxide (DMSO) at a stock concentration of 5 mM.

### Mice

Animal experiments were performed using C57BL/6J mice (SLC, Japan), which were handled in strict compliance with the guidelines for care and use of laboratory animals issued by the institutional ethical animal care committee of Kyungpook National University (Daegu, Korea). Mice were maintained under specific pathogen-free conditions.

### Cell culture and hypoxic conditions

HEK-293 human embryonic kidney epithelial cells (American Type Culture Collection, Manassas, VA, USA) were maintained in Dulbecco's modified Eagle's medium (DMEM, Hyclon, Logan, UT, USA) with 10% fetal bovine serum (FBS, Hyclon, Logan, UT, USA) and 1% antibiotics (100 units/mL penicillin, 100 mg/mL streptomycin, Invitrogen, Carlsbad, CA, USA). A549 adenocarcinomic human alveolar basal epithelial cells were maintained in Roswell Park Memorial Institute medium (RPMI) 1640 (Hyclon, Logan, UT, USA) containing 10% FBS and 1% antibiotics. Human umbilical vein endothelial cells (HUVECs, 4–10 passages) were grown on 1% gelatin-coated culture plates in Medium 199 (M199, Hyclon, Logan, UT, USA) supplemented with 20% FBS, 1% antibiotics, basic fibroblast growth factor (bFGF, 2 ng/mL), and heparin (5 unit/mL, Sigma, St Louis, MO). All cells were maintained at 37°C in an incubator with a humidified atmosphere of 5% CO_2_ and 95% air. For hypoxic condition, cells were cultured in hypoxic chambers (Thermo Scientific, Waltham, MA, USA and Astec, Fukuoka, Japan) at 5% CO_2_, with 1% O_2_ balanced with N_2_.

### MTT assay

Cells were seeded onto 96-well plates at a density of 5–10 × 10^3^ cells/well, and then incubated for 24 h. The media was removed, and the cells were treated with various concentrations of chemicals. After the cells were incubated for 24 h, 3-(4,5-dimethylthiazol-2-yl)-2,5-diphenyltetrazolium bromide (MTT, AMRESCO, Solon, OH, USA) solution was added to each well and incubated for another 4 h at 37°C. The resulting formazan crystals were dissolved in DMSO (100 μL/well), and the absorbance of the plate was read with a microplate reader (Infinite M200 Pro, TECAN, Männedorf, Switzerland) at 540 nm. Three replicate wells were used for each analysis.

### Dual luciferase assay

HEK-293 cells were seeded onto 96-well plates at a density of 5 × 10^3^ cells/well, and allowed to attach for 24 h. Next, cells were co-transfected with pGL3-HRE-luciferase plasmid containing five copies of HREs from human vascular endothelial growth factor (VEGF) genes, and pRL-SV40 plasmid encoding Renilla luciferase, using Vivamagic Transfection Reagent (Vivagene, Seongnam, Korea), according to the manufacturer's instructions. After transfection, cells were treated with each chemical compound for 24 h before reporter assay. Luciferase assays were performed using the Dual-Glo Luciferase Assay System (Promega, Madison, WI, USA), according to the manufacturer's instructions. Briefly, Dual-Glo Reagent was added with an equivalent volume of culture medium to each well, and the wells were incubated for 10 min. Firefly luminescence was measured using a microplate reader (Infinite M200 Pro, TECAN, Männedorf, Switzerland). Next, Dual-Glo Stop & Glo Reagent was added with an equivalent volume of culture mediumto each well, and the wells were incubated for 10 min. Renilla luminescence was measured using a microplate reader. Three replicate wells were used for each analysis, and the results were normalized to the activity of Renilla luciferase.

### Western blot analysis

Proteins extracted from cells using PRO-PREP (iNtRon Biotech, South Korea) were separated by SDS-PAGE and transferred to a nitrocellulose membrane (Whatman, Maidstone, UK). Membranes were blocked with 5% skim milk in Tris-buffered saline (TBS) containing 0.1% Tween-20 for 30 min at room temperature (RT). After blocking, membranes were incubated with specific primary antibodies overnight at 4°C, followed by incubation with horseradish peroxidase-conjugated mouse- or rabbit-IgG for 1 h at RT. Antibody binding was detected using an enhanced chemiluminescence (ECL) kit (BioRad, Hercules, CA) according to the manufacturer's instructions. Primary antibodies against the following factors were used: HIF-1α (BD Biosciences, San Diego, CA, USA), HIF-1β (Cell Signaling Technology, Danvers, MA, USA), β-actin (Santa Cruz Biotechnology, Santa Cruz, CA, USA), hydroxylated-HIF-1α (P564) (Cell Signaling Technology, Danvers, MA, USA), and PHD2 (Abcam, Cambridge, UK).

### Immunofluorescence assay

Cells were cultured on coverslips in four-well plates at a density of 1 × 10^5^ cells/well, and allowed to attach for 24 h. The media was removed; the cells were treated with various concentrations of chemicals, and the cells were incubated for 24 h. Cells were then fixed with 4% paraformaldehyde (PFA) solution and permeabilized with 1% Triton X-100. Fixed cells were blocked with 0.5% bovine serum albumin (BSA)/phosphate-buffered saline (PBS) and incubated with HIF-1α antibody (Santa Cruz Biotechnology, Santa Cruz, CA) overnight at 4°C. Alexa Fluor 488-conjugated anti-mouse IgG was used as the secondary antibody, and 4′,6-diamidino-2-phenylindole (DAPI) was used to label nuclei. Coverslips were mounted on slides, and cells were observed using fluorescence microscopy at 400X magnification (Carl Zeiss, AG, Germany).

### RNA isolation, reverse transcription-polymerase chain reaction (RT-PCR)

Total RNA was extracted from cultured cells using TRIzol Reagent (Invitrogen, Carlsbad, CA, USA), and cDNA was synthesized from total RNA using Moloney murine leukemia virus (MMLV) reverse transcriptase (Bioneer, Daejeon, Korea). The conditions for semiquantitative PCR were 30 cycles of: denaturation (94°C/30 s), annealing (50°C/40 s), extension (72°C/40 s), and final extension (72°C/10 min). Table 1 summarizes the sequences of the primers used in this study. Amplification real-time PCR was performed using a SYBR Green PCR Master Mix (Applied Biosystems, CA, USA). Each PCR reaction contained cDNA with ten-fold dilution and gene-specific primers. The thermal cycle used was 2 min at 50°C, 10 min at 95°C, and 40 cycles of 15 s denaturation at 95°C with 1 min annealing at 60°C. Mean cycle threshold (CT) values were calculated for gene expression, with normalization to β-actin or glyceraldehyde 3-phosphate dehydrogenase (GAPDH) as an internal control. Each experiment was performed in triplicate, and means were calculated.

**Table 1:** Primer sequences for PCR

name		Primer sequence
VEGF	Forward	5′-ACCATGAACTTTCTGCTC-3′
	Reverse	5′-GGACGGCTTGAAGATATA-3′
ALDOC	Forward	5′-TGAGCAGAAGAAGGAGTTGT-3′
	Reverse	5′-GGTCTCATGGAAGAAAATGA-3′
CA9	Forward	5′–GGAAGAAAACAGTGCCTATG-3′
	Reverse	5′–AGACCCCTCATATTGGAAGT-3′
GLUT1	Forward	5′–TACCCTGGATGTCCTATCTG-3′
	Reverse	5′–CACACAGTTGCTCCACATAC-3′
CXCR4	Forward	5′–GGCAGCAGGTAGCAAAGTGA-3′
	Reverse	5′–TGATGACAAAGAGGAGGTCG-3′
β-actin	Forward	5′–GACTACCTCATGAAGATC-3′
	Reverse	5′–GATCCACATCTGCTGGAA-3′

### Enzyme-linked immunosorbent assay (ELISA)

The amounts of VEGF protein secreted by the cells into the medium was determined with a VEGF ELISA kit, according to the manufacturer's instructions (#KHG0111, Invitrogen, Carlsbad, CA, USA).

### Bromodeoxyuridine (BrdU) cell proliferation assay

Human umbilical vein endothelial cells (HUVECs) were seeded onto 96-well plates at a density of 5 × 10^3^ cells/well, and allowed to attach for 24 h. The media was replaced with low serum medium (1% FBS in M199) for 16 h, and the cells were treated with various concentrations of chemicals for 24 h. Following incubation, cell proliferation was measured with a Cell Proliferation ELISA, BrdU kit (Roche, Mannheim, Germany), according to the manufacturer's instructions.

### *In vitro* cell migration assay

Migration of endothelial cells was tested using the Transwell system (8 μm pore size and 6.5 mm diameter) in 24-well plates (Corning Costar, Lowell, MA, USA). The lower sides of the filters were coated with 10 μL of type I collagen (0.5 mg/mL). Chemicals were added to the lower chamber in the presence of VEGF (20 ng/mL), and HUVECs (5 × 10^4^ cells/well) were seeded into the upper chamber in serum-free media. The chamber was incubated at 37°C for 24 h. Cells were then fixed with methanol, and stained with hematoxylin (Sigma, St Louis, MO, USA) and eosin (Sigma). Cells on the upper filter surface were removed, and migration was determined using a microscope, at 200× magnification, by counting cells that had migrated to the lower filter side. Samples were assayed twice, in triplicate.

### *In vitro* tube formation assay

A 96-well plate was coated with Matrigel (10 mg/mL, BD Biosciences, San Diego, CA, USA), which was then allowed to polymerize for 1 h at 37°C. HUVECs (3 × 10^4^ cells/well) were seeded on the surface of the Matrigel, with chemicals, in the presence of VEGF (20 ng/mL), at 37°C for 24 h. Changes in cellular morphology were observed under a microscope and photographed at 100×.

### Chick embryo chorioallantoic membrane (CAM) assay

Fertilized chicken eggs were maintained in a humidified incubator (Eunjo incubator company, Korea) at 37°C for three days. Chick eggshell membrane and 3–4 mL of egg albumin were removed from the egg. On Day 4.5, chemical-loaded Thermanox coverslips (NUNC, Rochester, NY, USA) were applied to the CAM surface. Two days later, 10% fat emulsion (Intralipid) was injected beneath the CAM and observed under a microscope. Retinoic acid (RA) was used as a positive control.

### Matrigel plug assay

Matrigel (200 μl, BD Biosciences) with DMSO, Compound 12 (100nM), and gefitinib (5 μM) were inoculated subcutaneously into C57BL/6J mice. Matrigel plugs were removed at 10 days after inoculation and photographed. To quantitate the formation of functional blood vessel, the content of hemoglobin was measured using the Drakin's reagent kit (Sigma,).

### *In vivo* tumor allogaft experiment and immunohistochemistry

LLC cells (1 × 10^6^) were injected subcutaneously into the flanks of 6-week-old C57BL/6J mice (Day 0)(SLC, Japan). Tumor size and body weight were measured 3 time a week. Tumor tissues were removed at day 20 after completion of injection of vehicle or drugs and fixed with 4% paraformaldehyde (PFA). After tissue processing for frozen sample preparation protocol, samples were embedded in OCT compound and frozen quickly by liquid nitrogen. Frozen blocks were cut into 10 μm sections and stained with hematoxylin and eosin (H&E) for light microscopy. Additional tissue sections for immunohistochemistry were blocked with 5% goat serum in PBST (0.03% Triton X-100 in PBS) and then incubated for 3 h at RT with following primary antibodies, anti-CD31 and anti-Ki67. To detect the hypoxic area in the tumors, Hypoxyprobe-1^™^ (60 mg/kg, Natural Pharmacia International) was iv injected 90 min before tissue fixation. Tumors were harvested, sectioned and stained with FITC-conjugated anti-Hypoxyprobe antibody.

### Statistical analysis

All data were expressed as mean ± standard deviations (SD) from at least 3 samples. Statistical comparisons were analyzed using the Student's *t*-test.

## SUPPLEMENTARY MATERIALS FIGURES


