# Supplementary material for: Molecular and functional evaluation of a novel HIF inhibitor, benzopyranyl 1,2,3-triazole compound
Source: Oncotarget. 2016 Dec 15;8(5):7801–13. doi: 10.18632/oncotarget.13955 (PMC5352362; doi:10.18632/oncotarget.13955)
Supplement: Supplementary file 1 [file oncotarget-08-7801-s001.pdf]

Molecular and functional evaluation of a novel HIF inhibitor, benzopyranyl 1,2,3-triazole compound

Supplementary Materials

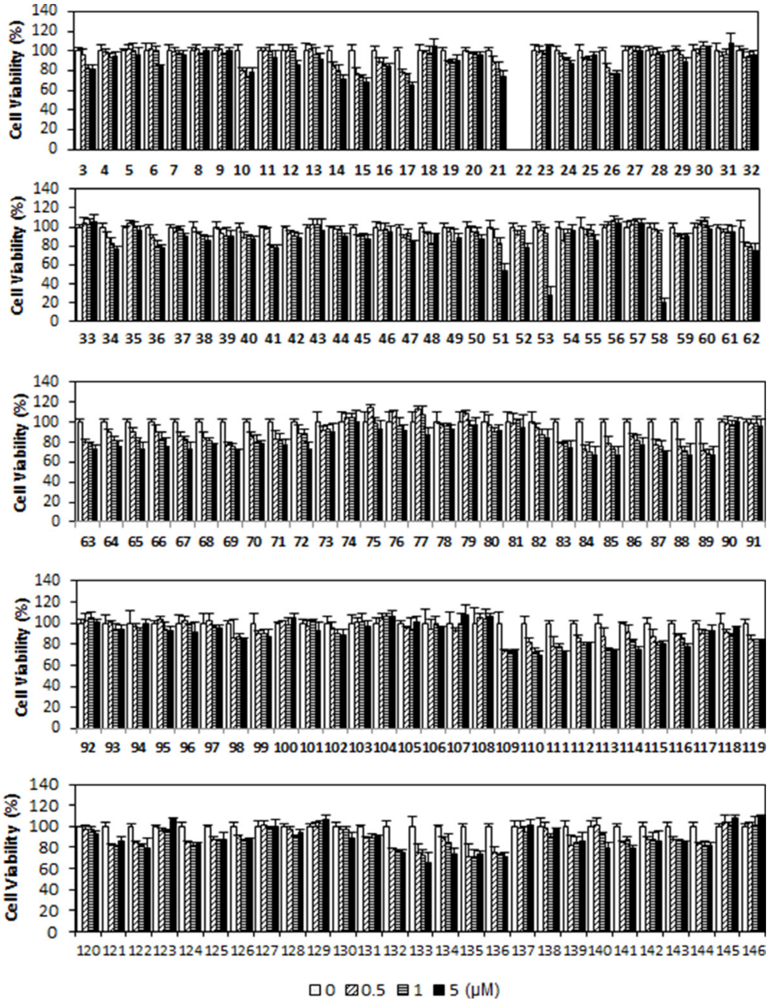

Supplementary Figure S1: Cytotoxicity of chemicals from house libraries. MTT Assay was performed with chemicals (Compound 3 to 146) at indicated doses in HEK -293 cells.

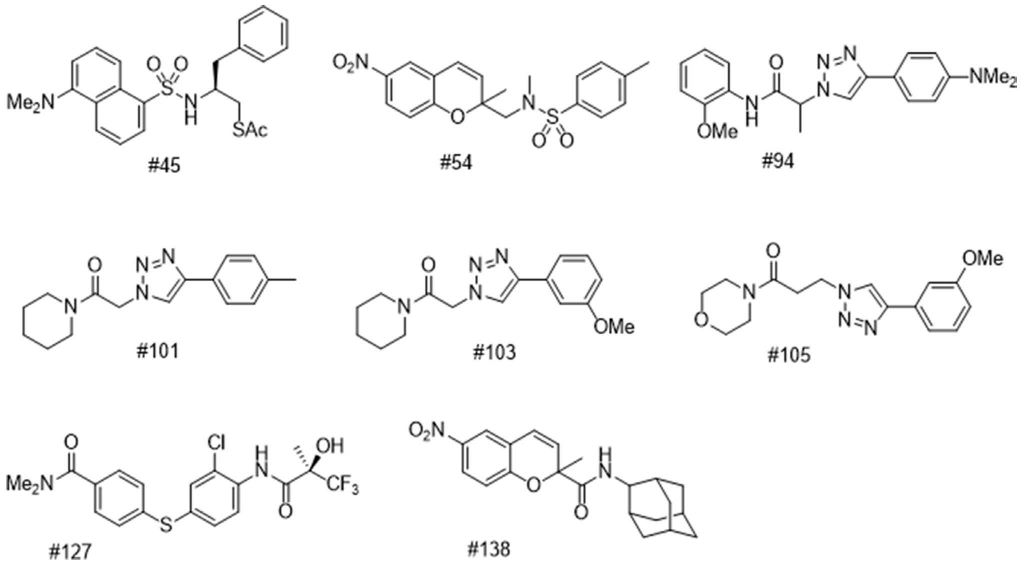

Supplementary Figure S2: Chemical structures of candidates for HIF-1α inhibitors identified both by HRE-luciferase activity (Figure 1) and western blot analysis in Figure 2.

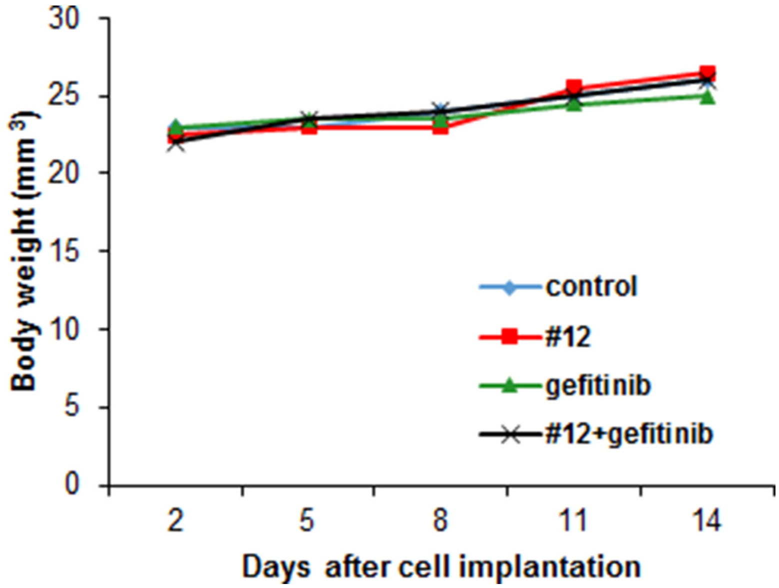

Supplementary Figure S3: The body weight of allograft mice as described in Figure 6A.
